# A Case of Viral Myocarditis Presenting With Acute Asthma Attack

**DOI:** 10.4021/jocmr823w

**Published:** 2012-05-15

**Authors:** Bunyamin Sertogullarindan, Bulent Ozbay, Hasan Ali Gumrukcuoglu, Mehmet Ata Akil, Mehmet Hakan Bilgin, Murat Yasar

**Affiliations:** a Department of Pulmonary Medicine, Medical Faculty, Yuzuncu Yil Universty, Van, Turkey; b Department of Cardiology Medical Faculty, Yuzuncu Yil Universty, Van, Turkey; c Department of Cardiology, Medicalpark Hospital, Van, Turkey; d Department of Otorhinolaryngology, Private Siirt Hospital, Siirt, Turkey

**Keywords:** Myocarditis, Asthma, Diagnosis

## Abstract

Acute viral myocarditis is one of the causes of heart failure. Cardiac asthma is commonly observed in elderly patients with left heart failure. If the pulmonary manifestations are prominent it can mask the involvement of heart. We report a young case of viral myocarditis mimicking acute asthma attack. Case Presentation: A 27-year-old young man with a history of asthma presented to the pulmonary department of our hospital with dyspnea, left sided chest pain, cough, wheezing. Asthma was diagnosed and treated, however his respiratory complaints have persisted. Laboratory evaluations revealed that elevated cardiac enzymes, Echocardiogram showed global hypokinesia in the left ventricle and a decrease of ejection fraction. We concluded that viral myocarditis can present itself like an acute asthma attack.

## Introduction

Myocarditis, which is an inflammatory process involving cardiac myocytes, can be induced by infections, immune-mediated damage, or toxins. It can be defined on the basis of histopathologic or clinical criteria [1]. Amongst the infectious causes, viral acute myocarditis is by far the most common [2]. In at least 10% of patients with viral infection, the virus may replicate in the heart [3]. Myocarditis generally results in a decrease in myocardial function, and this decrease may lead to pulmonary edema and congestive heart failure. Cardiac asthma is commonly observed in elderly patients with left heart failure. If the pulmonary manifestations are prominent it can mask the involvement of heart. We have reported a young case of myocarditis secondary to influenza a mimicking acute asthma attack for the first time.

## Case Report

**Figure 1:**
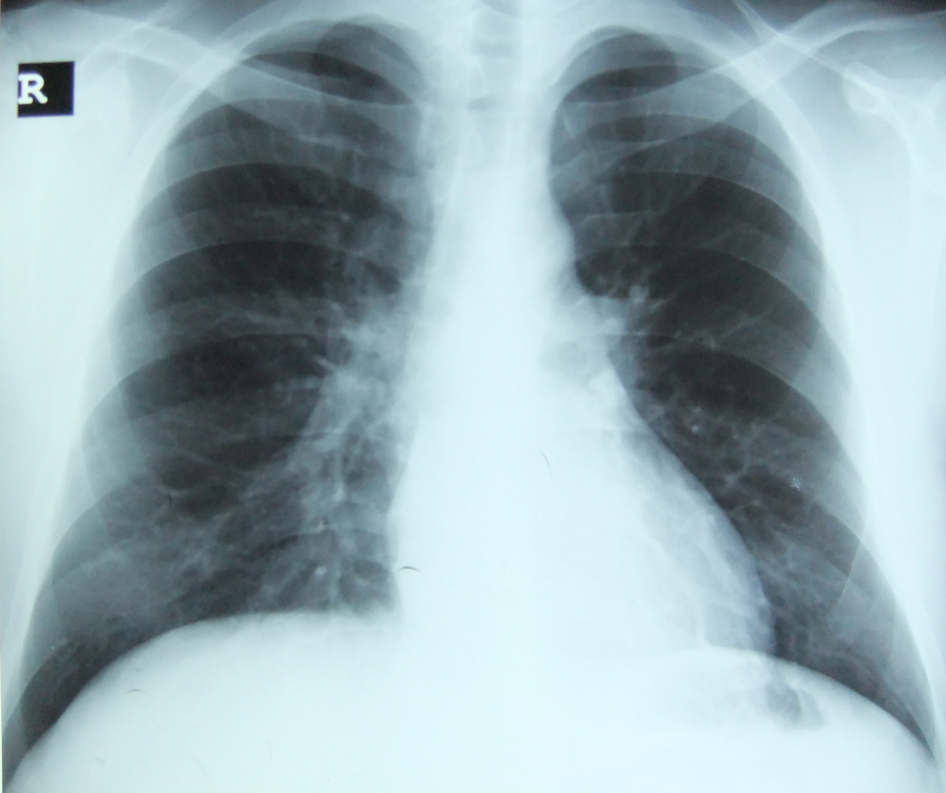
Chest radiography shows bronchovascular prominance on admission.

**Figure 2:**
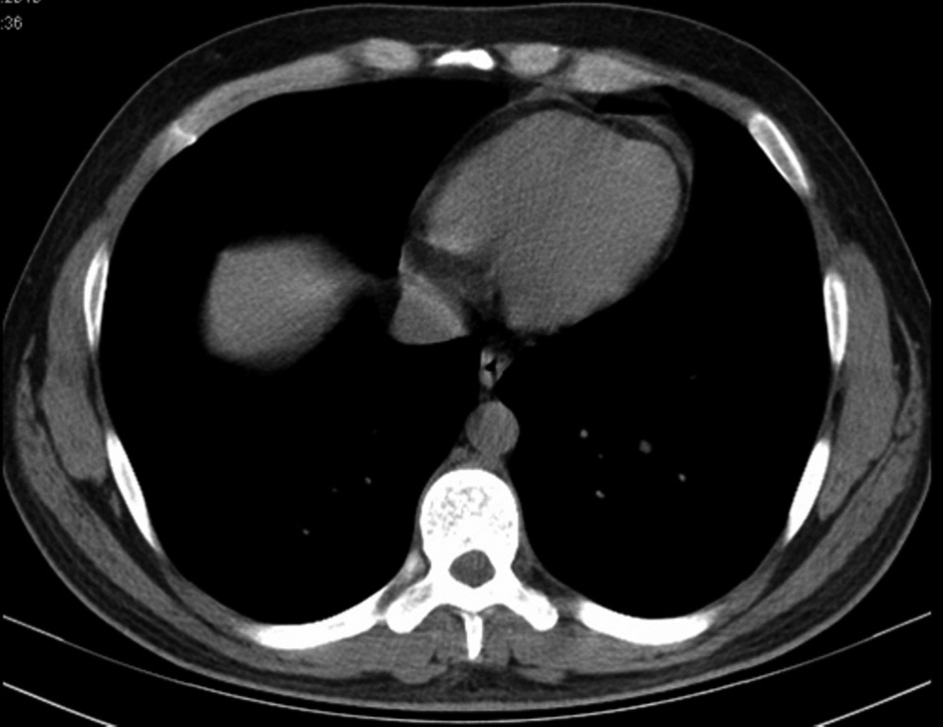
Chest tomography shows minimal pericardial effusion.

A 27-year-old young man presented to city hospital with 38 °C fever, dyspnea, cough, wheezing and left sided chest pain. His complaints began after an episode of viral infection one week before his admission. Influenza antigen test for influenza A and Ig E antibody was positive. His skin allergy test was positive to dust mite. Chest radiography showed bronchovascular prominence (Fig. 1). Chest computed tomography also revealed bronchovascular prominence and pericardial minimal effusion (Fig. 2). He was diagnosed with asthma and acute viral bronchitis and pericarditis. He was treated by oseltamivir, bronchodilator and anti-inflammatory, but his respiratuar compliants persisted. He was referred to our hospital two weeks later. His medical history revealed that his father had Wegener Diseases, and his mother had asthma. He has had symptoms of allergic rhinitis for two years but didn’t have diagnosis. He never smoked. On admission, the patient hemodynamic status was tachycardic as pulse 105 bpm and besides this, blood pressure was 110/70 mmHg, temperature 37 °C and respiratory rate 20/min. White blood cell count, erythrocyte sedimentation rate, C-reactive protein values were 21.7×10^9^/L, 25 mm/h and 7.9 mg/L respectively on admission. He had elevated cardiac enzymes as troponin I 0.01 μg/L, and CPK-MB 53 UI/L. C-ANCA was negative. Blood and sputum examination were all negative. Physical examination revealed that there were remarkable wheezes in both lungs and sinus tashicardia on auscultation. Electrocardiographic findings were nonspecific. Echocardiogram showed global hypokinesia in the left ventricular and a decrease of ejection fraction as 40%. After being started on diuretic and ACE inhibiteur therapy, the patient’s clinical condition significantly improved. One month later control Echocardiogram showed significantly improvement in left ventricular systolic function as 57%.

## Discussion

The clinical presentation of viral myocarditis varies from mild symptoms to acute hemodynamic compromise or sudden death [4]. Because of the variability of symptoms of acute viral myocarditis, the diagnosis can be often-overlooked. The onset of acute myocarditis starts on day 4 to 7 of the onset of viral symptoms [5]. Pandemic Influenza A (H1N1) related myocarditis has been found more frequent than the seasonal influenza outbreaks before [6]. There is an increased risk of myocarditis for Influenza patients, and this should not be ignored. Also acute myocarditis has been reported at follow for patients with pericarditis and Cytomegalovirus and Epstein-Barr virus infection [7, 8]. Therefore, patients with pericarditis and viral infection should be monitored more closely for myocarditis.

Myocarditis is one of the causes of acute congestive heart failure (CHF) [9]. CHF presenting with wheezing is termed cardiac asthma and it seems generally in elderly patients [10]. The wheezes are a manifestation of tracheobronchial edema and often are accompanied by overt signs of pulmonary edema [11, 12]. Cardiac asthma has been never reported in young patients with viral myocarditis. Asthma has a clinical diagnosis that includes episodic dyspnea and wheezing attacks. Asthmatic patients are generally younger than CHF patients. Asthma has a heritable component. A positive family history, allergic status and positive skin test are helpful for diagnostic guides [13]. Nonasthmatic patients with allergic rhinitis often have bronchial hyperresponsiveness and asthma develops most commonly in patients with rhinitis than in those without [14, 15]. The developments of bronchial asthma come out from a complex interaction of genetic predisposition and environmental causes with viral infection likely playing a significant role in the effect of environment on asthma inception [16]. The patient had most of the characteristics above, as expected, were diagnosed with asthma. However, patients did not respond to treatment. For this reason, patients diagnosed with asthma after influenza infection should be examined in terms of myocarditis. Especially in cases that do not respond to treatment, it is more important.

### Conclusion

Viral myocarditis can present itself like an acute asthma attack because of cardiac asthma and should be considered in the differential diagnosis of acute asthma attack in allergic patients with acute influenza infection. If there is compatible clinical picture, a recent viral infection should be considered in that it may cause acute myocarditis.

## References

[1] Bohn D, Benson L (2002). Diagnosis and management of pediatric myocarditis. Paediatr Drugs.

[2] Kuhl U, Pauschinger M, Seeberg B, Lassner D, Noutsias M, Poller W, Schultheiss HP (2005). Viral persistence in the myocardium is associated with progressive cardiac dysfunction. Circulation.

[3] Rezkalla SH, Kloner RA (2010). Influenza-related viral myocarditis. WMJ.

[4] Magnani JW, Dec GW (2006). Myocarditis: current trends in diagnosis and treatment. Circulation.

[5] Onitsuka H, Imamura T, Miyamoto N, Shibata Y, Kashiwagi T, Ayabe T, Kawagoe J (2001). Clinical manifestations of influenza a myocarditis during the influenza epidemic of winter 1998-1999. J Cardiol.

[6] Ukimura A, Izumi T, Matsumori A (2010). A national survey on myocarditis associated with the 2009 influenza A (H1N1) pandemic in Japan. Circ J.

[7] Roubille C, Brunel AS, Gahide G, Vernhet Kovacsik, Le Quellec A (2010). Cytomegalovirus (CMV) and acute myocarditis in an immunocompetent patient. Intern Med.

[8] Schultz JC, Hilliard AA, Cooper LT, Jr (2009). , Rihal CS. Diagnosis and treatment of viral myocarditis. Mayo Clin Proc.

[9] Massie BM, Goldman L, Ausiello D (2003). Heart Failure: Pathophysiology And Diagnosis. Cecil Medicine.

[10] Fishman AP (1989). Cardiac asthma--a fresh look at an old wheeze. N Engl J Med.

[11] Sasaki F, Ishizaki T, Mifune J, Fujimura M, Nishioka S, Miyabo S (1990). Bronchial hyperresponsiveness in patients with chronic congestive heart failure. Chest.

[12] Faggiano P (1994). Abnormalities of pulmonary function in congestive heart failure. Int J Cardiol.

[13] Global Initiative for Asthma Global Strategy for Asthma Management and Prevention. 2010 Revision. http://www.ginasthma.org/guidelines-gina-report-globalstrategy-for-asthma.html. Date updated: December 2010. Date accessed: 21 June2011..

[14] Koh YY, Lee MH, Kim CK, Min YG, Kim YK, Min KU, Kim YY (1998). A familial predisposition in bronchial hyperresponsiveness among patients with allergic rhinitis. J Allergy Clin Immunol.

[15] Wright AL, Holberg CJ, Martinez FD, Halonen M, Morgan W, Taussig LM (1994). Epidemiology of physician-diagnosed allergic rhinitis in childhood. Pediatrics.

[16] Dulek DE, Peebles RS Jr Viruses and asthma. Biochim Biophys Acta. 2011 Feb 1. Available from: URL: http://www.ncbi.nlm.nih.gov/pubmed/21291960.

